# Mapping online hate: A scientometric analysis on research trends and hotspots in research on online hate

**DOI:** 10.1371/journal.pone.0222194

**Published:** 2019-09-26

**Authors:** Ahmed Waqas, Joni Salminen, Soon-gyo Jung, Hind Almerekhi, Bernard J. Jansen

**Affiliations:** 1 University of Liverpool, Liverpool, United Kingdom; 2 CMH Lahore Medical College & Institute of Dentistry, Lahore, Pakistan; 3 Qatar Computing Research Institute, Hamad Bin Khalifa University, Doha, Qatar; 4 Turku School of Economics at the University of Turku, Turku, Finland; 5 Hamad Bin Khalifa University, Doha, Qatar; University of Toronto, CANADA

## Abstract

Internet and social media participation open doors to a plethora of positive opportunities for the general public. However, in addition to these positive aspects, digital technology also provides an effective medium for spreading hateful content in the form of cyberbullying, bigotry, hateful ideologies, and harassment of individuals and groups. This research aims to investigate the growing body of online hate research (OHR) by mapping general research indices, prevalent themes of research, research hotspots, and influential stakeholders such as organizations and contributing regions. For this, we use scientometric techniques and collect research papers from the Web of Science core database published through March 2019. We apply a predefined search strategy to retrieve peer-reviewed OHR and analyze the data using CiteSpace software by identifying influential papers, themes of research, and collaborating institutions. Our results show that higher-income countries contribute most to OHR, with Western countries accounting for most of the publications, funded by North American and European funding agencies. We also observed increased research activity post-2005, starting from more than 50 publications to more than 550 in 2018. This applies to a number of publications as well as citations. The hotbeds of OHR focus on *cyberbullying*, *social media platforms*, *co-morbid mental disorders*, and *profiling of aggressors and victims*. Moreover, we identified four main clusters of OHR: (1) *Cyberbullying*, (2) *Sexual solicitation and intimate partner violence*, (3) *Deep learning and automation*, and (4) *Extremist and online hate groups*, which highlight the cross-disciplinary and multifaceted nature of OHR as a field of research. The research has implications for researchers and policymakers engaged in OHR and its associated problems for individuals and society.

## Introduction

The advent of the modern Internet opens doors to a plethora of positive opportunities for the general public. These opportunities span across equity in education and general access to knowledge, modes of entertainment, consumerism, and e-participation. However, in addition to these positive aspects, digital technology also provides an effective medium for spreading hateful content in the form of bigotry and hateful ideologies, as well as cyberbullying and harassment of individuals and groups on social media platforms [1,2]. Online hate, albeit conducted in the virtual world, may have dire real-life consequences at both individual and population levels. For example, the cyberbullying among youth and student populations and subsequent links with poor mental health, depression, trauma, substance misuse, and a higher risk of suicide are well-documented [3–6]. Recent estimates have placed exposure to online hate ranging from 31% to 67% across different study samples [7]. Among New Zealanders, for example, 11% of adults have been personally targeted by online hate [1], whereas, in the US, 41% of adults have experienced online hate speech and harassment [8]. Online hate has been shown to predominantly target and influence minorities, young age groups, people with disabilities, and the LGBTQ (Lesbian, Gay, Bisexual, Transgender, Queer) community [1].

Online hate spreading has also emerged as a tool for politically motivated bigotry, xenophobia, homophobia, and excessive nationalism [9–12]. An example can be seen in the 2016 US elections; the narrative of “Make America Great Again” has empirically been shown to have amplified the online presence of white supremacists [9]. Social media platforms have granted a new spirit to radical nationalist groups including Klansmen and Neo-Nazis by ensuring anonymity or pseudonymity (i.e., disguised identity), ease of discussions, and spread of radical ideologies [1]. Moreover, social media and online forums have provided hate-driven terrorist groups a medium for launching propaganda to radicalize youth globally [13]. These groups use images and Internet videos to communicate their hateful intent, to trigger panic, and to cause psychological harm to the general public [14]. As a prime example of cyberterrorism, the Islamic State of Iraq & Syria (ISIS) effectively used social media to recruit youngsters from Europe to participate in the Syrian conflict [12]. Their social media campaigns led to at least 750 British youngsters joining Jihadi groups in Syria [13]. Overall, these real-world phenomena highlight the very real negative impact of spreading online hate and suggest that online hate can be considered as a major public concern.

However, online hate is a complex phenomenon—with its definition depending on theoretical paradigms, disciplines, and forms of victimization [1,15]. Due to this complexity, online hate research (OHR) is a fragmented field with a growing number of research papers across disciplines, as the adverse effects of online hate are more widely recognized in society and as new disciplines (e.g., computer science, psychology) are introducing their own approaches to study and solve the associated problems. Due to this increasing body of research, there is a need for literature analyses that map the current state of OHR. While several evidence-synthesis approaches have attempted to summarize and critically review the literature on online hate, these tend to be based on heterogeneous methodologies and restricted to a particular discipline or field of study [9,10,23,13,16–22]. For example, an elaborate effort by the British Institute of Human Rights sought to systematically map studies about initiatives against cyberbullying and inform legislative efforts by the European Union [21]. A qualitative approach by Awan sought to provide evidence regarding the use of social media platforms by ISIS by examining 100 Facebook pages and 50 Twitter users [13]. Country-specific efforts included Gagliardone et al.’s efforts to map politically driven online hate in Ethiopia by reviewing relevant Facebook profiles, pages, and groups with more than 100 followers [23], which provided a framework for analyzing online hate speech and explored the continuum between freedom of expression [23]. Cyber-bullying has also attracted attention from public health and mental health professionals. Most influential and cited work in this domain is attributed to Tokunaga, who critically reviewed and synthesized evidence on cyberbullying victimization [20].

However, none of the previous work, to the knowledge of authors, has focused on the mapping of general research indices, prevalent themes of research, research hotspots, and influential stakeholders such as organizations and contributing regions regarding OHR. This undertaking is essential as such analyses help to evaluate the field-specific impact of scholarly research, as well as the impact of scientists, collaborative networks, and institutes. Therefore, we set out to map OHR using scientometric analysis, defined as the “quantitative study of science, communication in science, and science policy” [24]. Most importantly, scientometrics helps identify influential research studies resulting in the progress and evolution of a specific field of science [24]. By using reproducible statistical techniques, stakeholders can quantize the research output, citation rates, influential funding agencies, journals, scientists, institutes, and regions involved in the progress of the scientific discipline [24]. By mapping these trends, researchers, policymakers, and funding agencies can determine areas where an increase or restriction in research work and funding is required [25–27]. Therefore, this investigation aims to address this paucity of data using advanced scientometric techniques.

## Methodology

### Search strategy

We defined the focal topic of study as online hate. We identified several definitions from the prior literature that helped us understand the nature of the phenomenon and to collect a list of concepts that reflect the multifaceted nature of OHR. Definitions of online hate vary, but a unifying factor is the use of technology for expressions that are harmful to individuals, groups, or society as a whole. An example of a definition that encompasses this duality is that of Kaakinen et al., according to whom online hate has two defining characteristics: it is *technology-mediated* and *intends to offend*, discriminate and abuse a person or a group based on group defining characteristics such as gender, race, nationality, ethnicity, disability, or sexual orientation [7].

In the course of exploring the definitions, we compiled a list of keywords for the electronic search carried out to identify the body of research about OHR (see Table 1).

**Table 1:** Key concepts in online hate research, operationalized as search terms.

Concept	Definition
Online hate	Forms of hateful expressions disseminated on the Internet, typically targeting a specific group or individual
Online hate speech	As above, but fulfilling the legal definition of hate speech (that may vary by country)
Online toxicity	Social media commenting that is likely to reduce an individual’s desire to participate in discussions due to fear of being ridiculed
Online abusive language	Use of slurs and vocabulary that is offensive to other Internet users
Cyberbullying	Systematically attacking a person or people via electronic channels; e.g., name calling, discrediting, shaming
Online harassment	Predatory and oppressive behavior on the Internet; e.g., sending sexual messages to non-consenting individuals
Online firestorms	Inflammatory forms of online discussions (“fighting”), usually taking place in discussion forums between rivaling groups

In addition to operationalizing the concepts in Table 1 as search terms, we defined a list of popular social media platforms that were also used as search terms, as several studies focus on hate taking place in a specific social media platform. Using the Web of Science core database, an electronic search was conducted to retrieve peer-reviewed research studies (published through March 2019) pertaining to online hate. Overall, this search strategy encompassed important concepts pertaining to online hate and popular platforms: “TS = (Hate OR toxicity OR cyberbullying OR bullying OR harass* OR firestorm* OR abuse OR abusive OR ‘abusive language’ OR maltreat* OR oppress* OR persecut* OR taunt* OR bully* OR bullies OR victim* OR ‘hate speech’) AND TI = (Online OR ‘social media’ OR web OR virtual OR cyber OR Orkut OR Twitter OR facebook OR Reddit OR Instagram OR snapchat OR youtube OR whatsapp OR wechat OR QQ OR Tumblr OR linkedin OR pinterest)”. As mentioned, this search strategy was formulated based on an initial reading of the literature and identifying commonly emerging terms in the studies about online hate. No restrictions were applied for year of publication or language.

The search process resulted in a total of 3,371 research articles for a scientometric analysis. The data curated from the Web of Science (core database) included the citation characteristics, citation counts, and cited references. The Web of Science core database is one of the most frequently used databases for scientometric analyses. It was chosen primarily because it indexes detailed citations and full records of cited references that help in elucidating co-citation relationships between related documents [28].

### Operational definitions and inclusion criteria

The present mapping study is a broad overview of OHR. In line with our objectives, a broader interpretation of online hate was preferred, covering all forms of expressions that spread, incite, promote, or justify hate against groups or individuals [21]. This interpretation was adapted from the framework for online hate proposed by the British Institute of Human Rights [21]. All forms of expressions on a macro-level including racial hatred, xenophobia, anti-Semitism, aggressive nationalism, and hatred against minorities and migrants were included. On an individual level, various forms of expression, for instance, partner abuse as well as cyber-bullying against school children owing to their racial, ethnic, sexual background, and disabilities were included [21]. We acknowledge that there are alternative definitions for online hate and online toxicity, the latter of which can be defined as rude, disrespectful, or unreasonable commenting that is likely to make one leave a discussion [29,30]. Most of these definitions perceive online hate as a conceptually broad phenomenon that touches many stakeholder groups. For that reason, we consider broad inclusion criteria to be relevant for this research.

### Co-citation analysis and knowledge mapping

In the first phase, data curated from the Web of Science core database (WOS) was utilized for knowledge mapping based on the theory of document co-citation. According to this theory, when two documents are co-cited by one document, they are connected in a co-citation relationship [31].

Co-citation analyses were performed using CiteSpace software (n = v4.0, Drexel University, Pennsylvania, US). The bibliographic records retrieved from WOS were fed into the CiteSpace software, and “sliced” into three-year slices, where each slice was represented by 50 documents with the highest cited frequency. Titles, abstracts, and keywords were used as terms sources while cited references were used as nodes.

After that, network analysis was run using pathfinder network scaling while allowing for the pruning of sliced networks [25–27]. All bibliographic data were then visualized as merged and static networks/clusters. Articles were represented as nodes, while the relationship between nodes was visualized as lines or edges. Two important matrices were used to demonstrate the overall structural properties of the network: modularity and silhouette value. Note that a high value of modularity (close to 1) corresponds to a good network structure that is reasonably divided into loosely coupled clusters, and a high silhouette score represents an appropriately homogenized cluster. This technique allowed for the visualization of important publications in a collaborative network based on their centrality values, also identified as a tree ring representing their history of citations and year-wise patterns [25–27]. New theories and landmark studies with high between-ness centrality were identified as purple rings while citation bursts were visualized as red tree rings [25–27].

Citation bursts were defined as articles attracting significant research activity in a given period. Clusters and themes of research in this field were identified by running a cluster analysis that identified the publication record cited in a specific set of publications, and the clusters were named using naming algorithms including TF*IDF; Mutual Information (MI) and Log Likelihood Ratio (LLR) [25–27]. Each cluster was also depicted by a year representing the mean year of publications of all included research studies. Out of these methods, LLR has been shown to be the most accurate [25–27]. The first method, TF*IDF, utilizes terms that are weighted by term frequencies (TF) multiplying inverted document frequencies (IDF) [25–27]. Log-likelihood ratio tests choose the most appropriate clustering label by assessing the strength of the bond between a term and the cluster [25–27]. Generally, the higher the LLR, the better the evidence. Lastly, the mutual information method is used for feature selection in machine learning; however, it works better with larger datasets [25–27].

## Results

### Research activity

**Fig 1:**
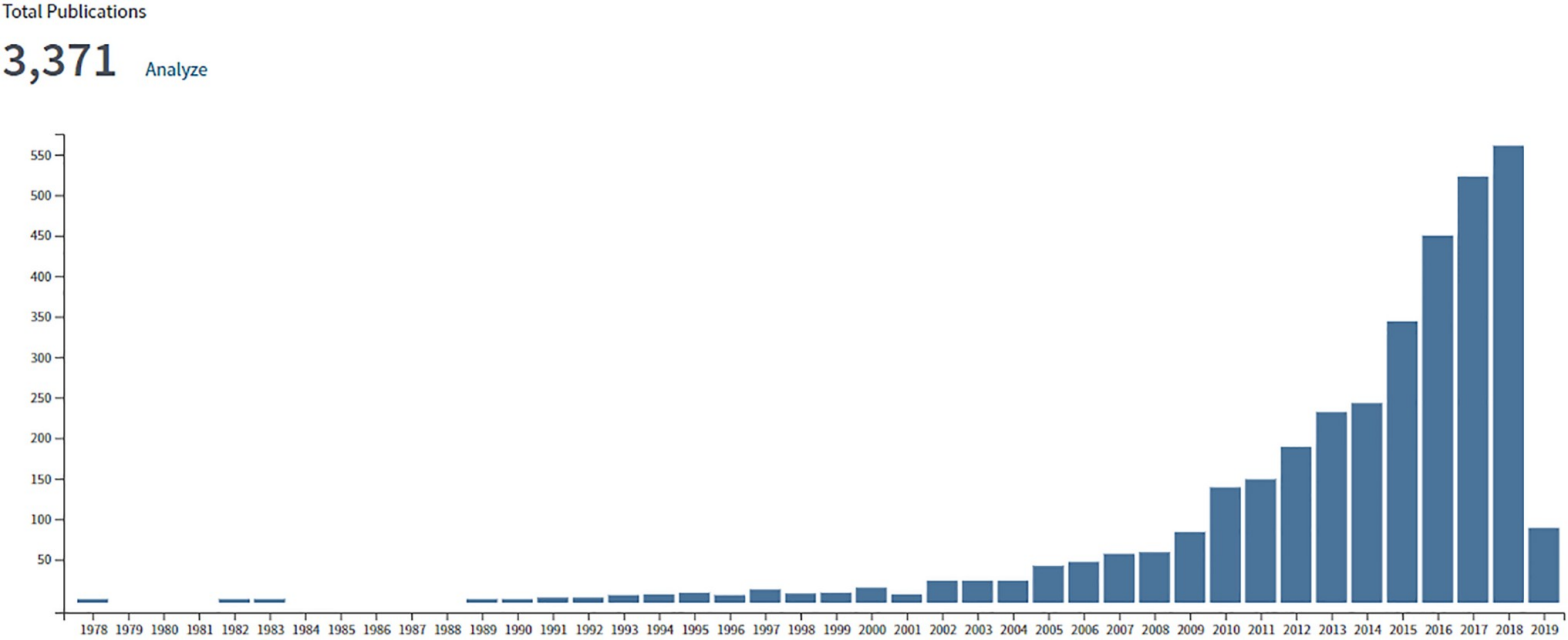
Rate of publications from the year 2000 to 2018.

**Fig 2:**
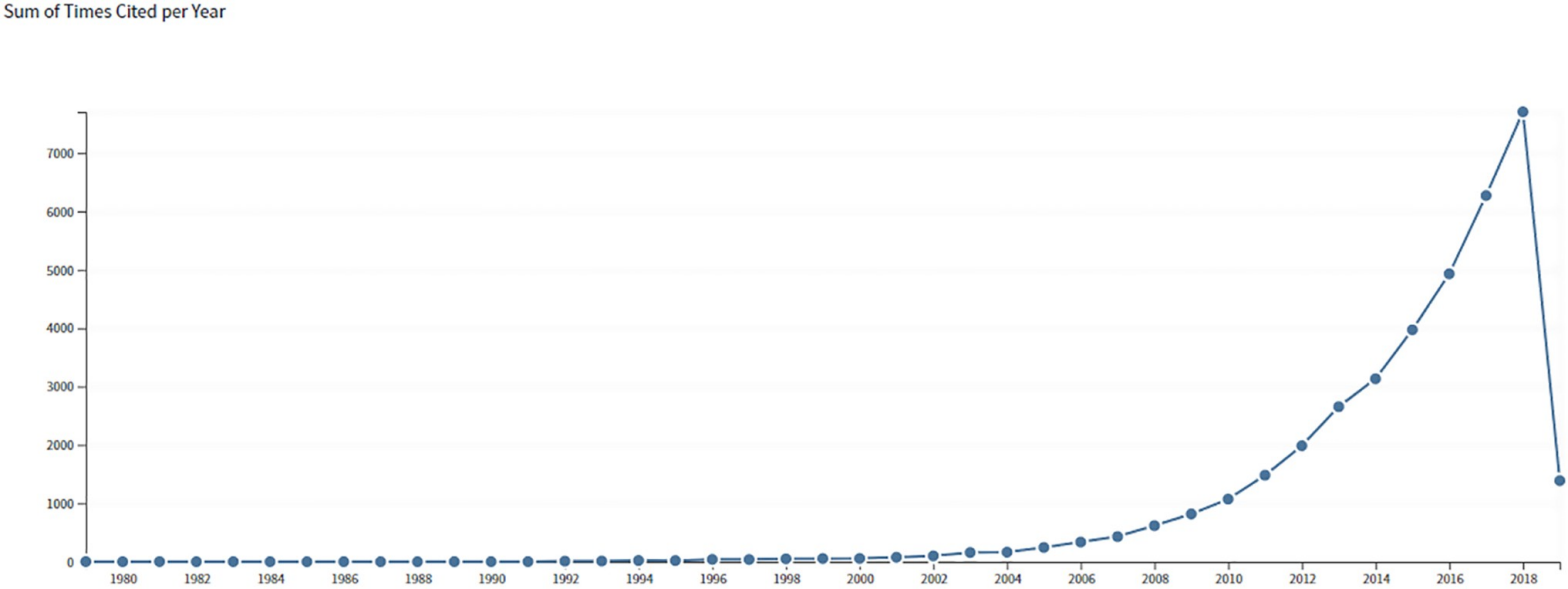
Rate of citations from the year 2000 to 2018.

The search process yielded a total of 3,371 publications that were included in the scientometric analysis. These publications boasted an h-index of 82, 11.23 citations per item, cited for a total of 37,848 times overall (n = 33,721 excluding self-citations). Increased publication and citation activities were observed post- 2005 starting from >50 publication to > 550 in the year 2018 (Figs 1 and 2).

### Top organizations, funders, and regions

**Fig 3:**
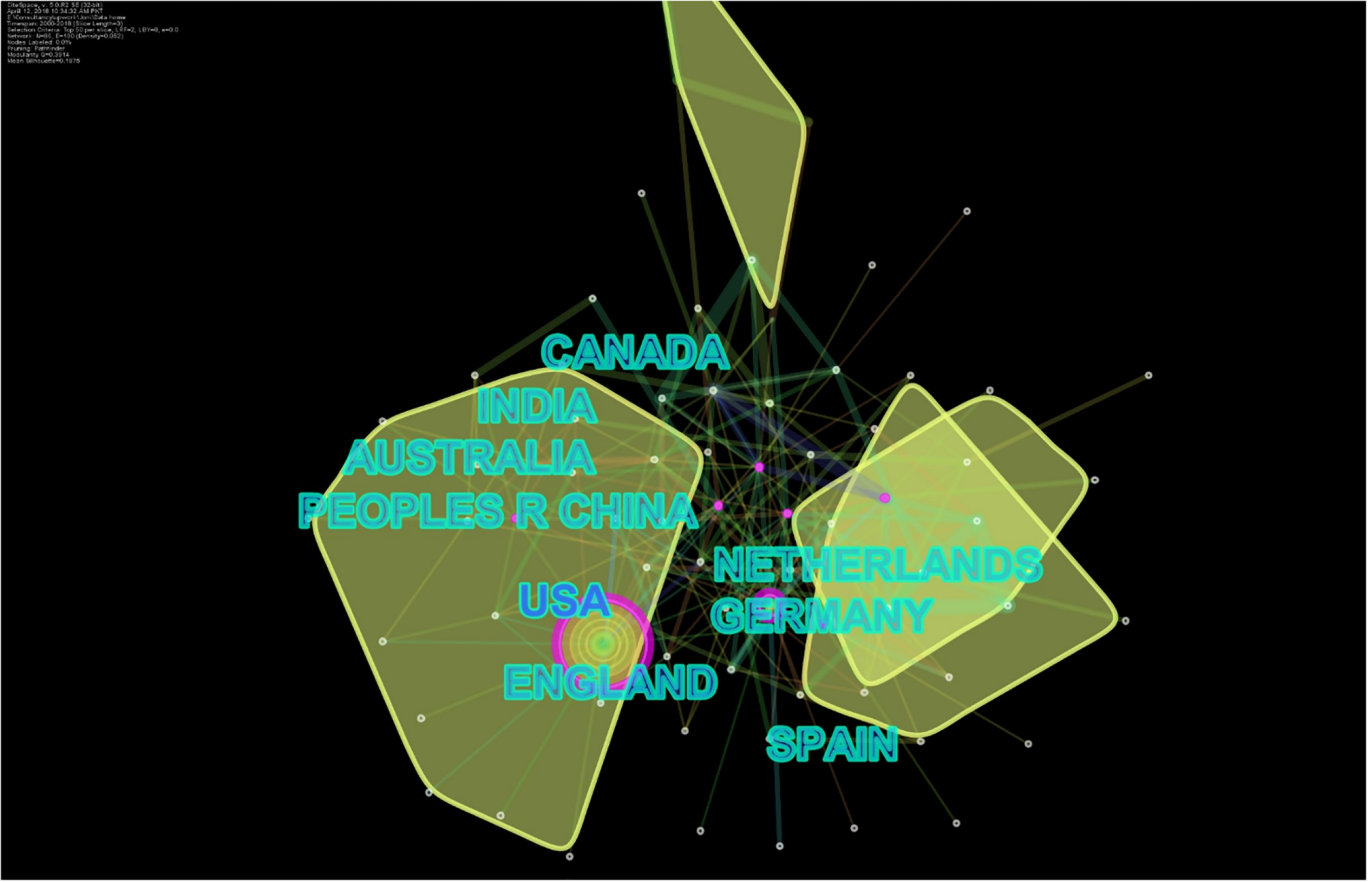
Collaborative networks based on countries.

**Fig 4:**
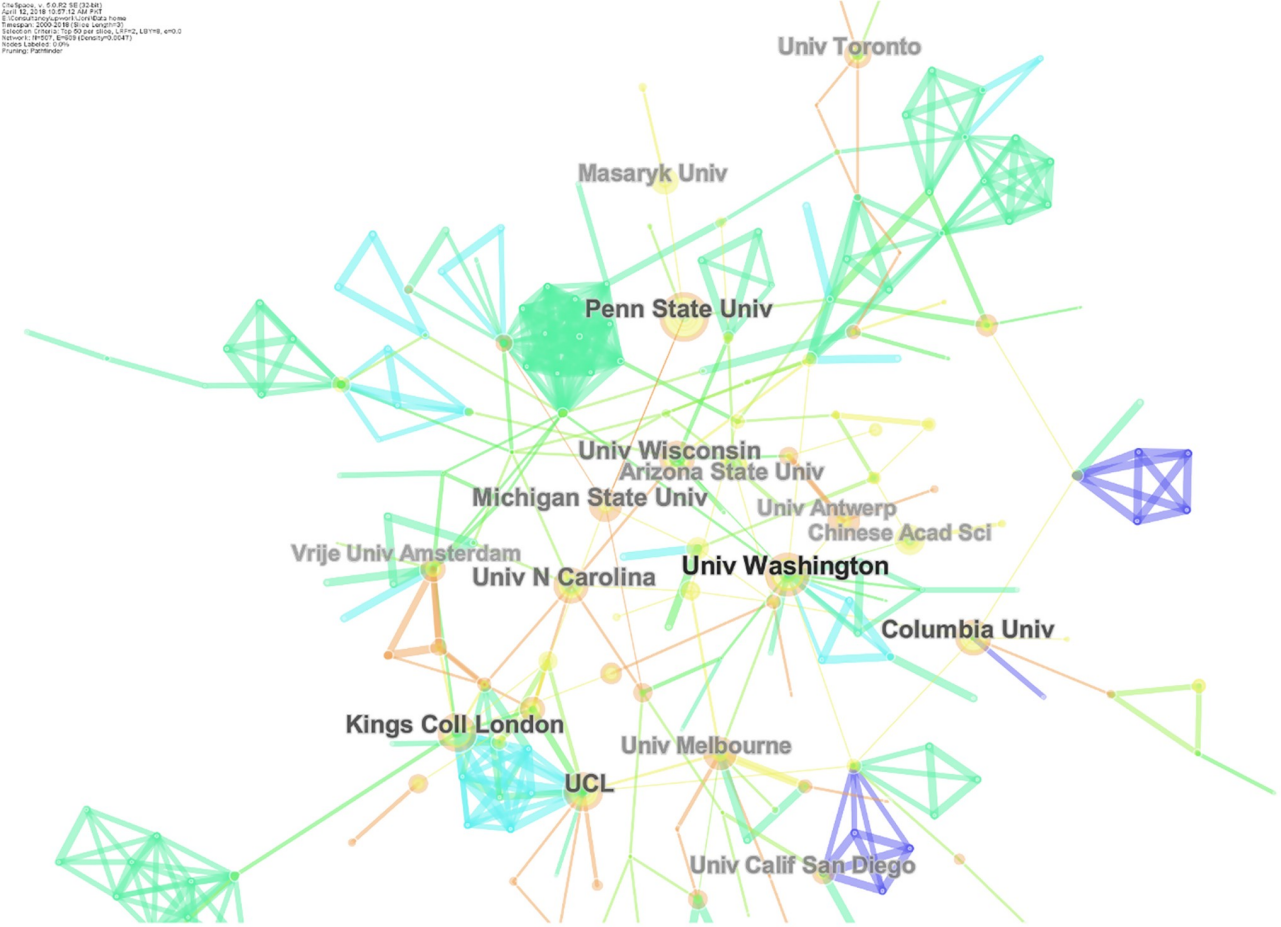
Collaborative networks of institutes.

The United States of America (US) was the most frequent publisher in this domain with 1,205 publications, followed by England, Australia, China, Canada, India, Germany, Spain, the Netherlands, and Italy. Among universities, the University of London, UK was the most frequent contributor, followed by university systems in the US: the University of California System, the Pennsylvania Commonwealth System of Higher Education, State University of Florida, the University of North Carolina, the University of Texas System, the University of Georgia, the University of Washington, Columbia University, and the University of Washington in Seattle. Top funders included United States Department of Health and Human Services (HHS)/National Institutes of Health (NIH), National Natural Science Foundation of China, National Science Foundation, Economic and Social Research Council, National Institute of Drug Abuse, European Union, and Catalan Institution for Research and Advanced Studies (ICREA). Collaborative networks of countries and institutes are presented as Figs 3 and 4, while frequencies of publications by top countries are presented in Table 2.

**Table 2:** Top countries, institutes, and sources according to the number of publications.

Country	n	Institute	n	Journal	n	Conference	n
**USA**	**1,205**	University of London, UK	**70**	Computer in Human Behavior	**76**	IEEE ACM International Conference on Advances in Social Network Analysis and Mining	**14**
**England**	**317**	**University of California, USA**	**68**	Lecture Notes in Computer Science	**46**	Annual International Conference on Education Research and Innovation	**4**
**Australia**	**194**	Pennsylvania Commonwealth System of Higher Education	**50**	Cyberpsychology, Behavior & Social Networking	**36**	International Conference on World Wide Web	**4**
**China**	**179**	State University of Florida	**48**	Journal of Medical Internet Research	**32**	ACM Conference on Computer Supported Cooperative Work and Social Computing	**4**
**Canada**	**171**	University of North Carolina System	**36**	Journal of Adolescent Health	**25**	Saudi Computer Society National Computer Conference	**3**
**India**	**169**	University of Texas System	**35**	Journal of Youth and Adolescence	**23**	IEEE International Conference on Trust Security and Privacy in Computing and Communication Trustcom	**3**
**Germany**	**145**	University of Georgia	**33**	Procedia Social and Behavioral Sciences	**21**	ACM SIGSAC Conference on Computer And Communications Security	**3**
**Spain**	**136**	University of Washington	**32**	PloSOne	**23**	International Conference on Intelligence and Security Informatics Cybersecurity and Big Data	**3**
**Netherlands**	**99**	Columbia University	**31**	New Media Society	**20**	**-**	
**Italy**	**96**	University of Washington in Seattle	**31**	Child Abuse & Neglect	**18**	**-**	

### Top sources

Top sources included Computer in Human Behavior, Lecture Notes in Computer Science, Cyberpsychology, Behavior & Social Networking, Journal of Medical Internet Research, Journal of Adolescent Health, Journal of Youth and Adolescence, Procedia Social and Behavioral Sciences, PLOS One, New Media Society, and Child Abuse & Neglect. While most frequent conference proceedings were published by IEEE ACM International Conference on Advances in Social Network Analysis and Mining, Annual International Conference on Education Research and Innovation, International Conference on World Wide Web, ACM Conference on Computer Supported Cooperative Work and Social Computing, Saudi Computer Society National Computer Conference, IEEE International Conference on Trust Security and Privacy in Computing and Communication Trustcom, ACM SIGSAC Conference on Computer and Communications Security and International Conference on Intelligence and Security Informatics Cybersecurity and Big Data. Frequencies of publications by top sources are presented in Table 2.

### Fields of publication

Top ten fields of publication included computer science information systems (n = 325), computer science theory methods (n = 282), criminology (n = 263), communication (n = 221), multidisciplinary psychology (n = 193), electrical/electronic engineering (n = 187), computer science interdisciplinary publications (n = 183), psychiatry (n = 168), educational research (n = 180) and clinical psychology (n = 154).

### Top papers based on centrality in respective clusters

**Fig 5:**
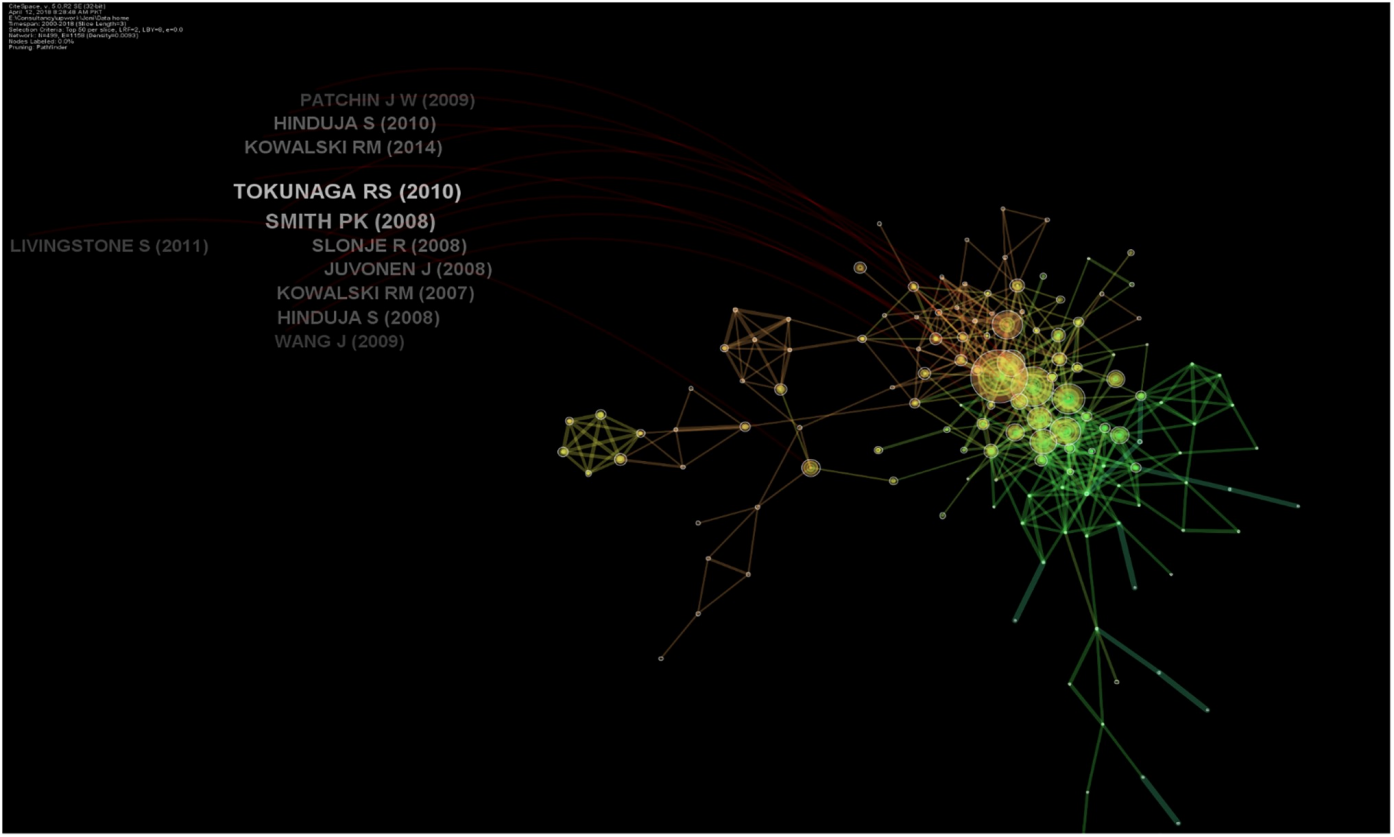
Influential authors in online hate.

Top papers were judged based on their values of centrality, where a value of 0.1 indicates a central publication. In a collaborative and co-cited network of publications, a high centrality value reflects highly significant research studies. However, in this analysis, none of the studies reached a centrality value of 0.1, indicating no central publication in the respective cluster. However, top centrality value (> 0.01) was achieved by 14 studies (Table 3 and Fig 5). The majority of these papers focused on cyberbullying among adolescents. Tokunaga RS (2010) and Kowalski RM (2007) were found to be most central to entities with centrality values of 0.04.

**Table 3:** Top articles based on centrality values.

Citations in WOS Core	Burst years*	Centrality	Sigma	Author	Year	Source	Cluster
152	8.84	0.04	1.39	Tokunaga RS	2010	Comput Hum Behav	2
77	19.59	0.04	2.14	Kowalski RM	2007	J Adolescent Health	1
122	20.5	0.03	1.99	Smith PK	2008	J Child Psychol Psyc	1
86	11.35	0.03	1.34	Slonje R	2008	Scand J Psychol	1
44	14.39	0.03	1.52	Raskauskas J	2007	Dev Psychol	1
41		0.03	1	Calvete E	2010	Comput Hum Behav	2
7	4.06	0.03	1.15	Ybarra ML	2007	J Adolescent Health	1
84	3.04	0.02	1.07	Hinduja S	2010	Arch Suicide Res	1
80	12.59	0.02	1.31	Juvonen J	2008	J School Health	1
46	3.86	0.02	1.09	Erdur-baker O	2010	New Media Soc	2
45	9.01	0.02	1.15	Dehue F	2008	Cyberpsychol Behav	1
27	7.34	0.02	1.15	Zweig JM	2013	J Youth Adolescence	10
20	5.74	0.02	1.13	Mitchell KJ	2007	Am J Prev Med	7
7		0.02	1	Borrajo E	2015	Comput Hum Behav	10
84	19.02	0.01	1.23	Kowalski RM	2014	Psychol Bull	2
59	9.05	0.01	1.08	Livingstone S	2011	Risks Safety Interne	6
53	16.98	0.01	1.09	Patchin JW	2006	Youth Violence Juv J	1
41	13.3	0.01	1.11	Li Q	2006	School Psychol Int	1
38	8.47	0.01	1.11	Kowalski RM	2013	J Adolescent Health	2
34	12.55	0.01	1.09	Williams KR	2007	J Adolescent Health	1
33	4.86	0.01	1.04	Mesch GS	2009	Cyberpsychol Behav	6
32	8.98	0.01	1.06	Reyns BW	2011	Crim Justice Behav	10
30	9.57	0.01	1.11	Ybarra ML	2007	J Adolescent Health	1
28	6.22	0.01	1.08	Gamez-guadix M	2013	J Adolescent Health	2
27	6	0.01	1.03	Bauman S	2013	J Adolescence	2
25	7.19	0.01	1.09	Ybarra ML	2007	Arch Pediat Adol Med	7
22	8.06	0.01	1.05	Beran T	2005	Journal Of Educational Computing Research	1
10		0.01	1	Kloess JA	2014	Trauma Violence Abus	6
9		0.01	1	Mitchell KJ	2011	J Adolescent Health	6
8		0.01	1	Montiel I	2016	Child Abuse Neglect	6
8	4.09	0.01	1.02	Mitchell KJ	2007	J Adolescent Health	7
7		0.01	1	Perren Sonja	2010	Child Adolesc Psychiatry Ment Health	2
6		0.01	1	Staude-muller F	2012	Eur J Dev Psychol	6
6		0.01	1	Reyns BW	2012	Deviant Behav	10
6	3.67	0.01	1.02	Mitchell KJ	2003	Youth Soc	1
6		0.01	1	Livingstone S	2010	New Media Soc	6
5	3.06	0.01	1.03	Fleming MJ	2006	Youth Soc	7
5	3.06	0.01	1.02	Erdur-baker O	2007	J Euroasian Ed Res	1
4		0.01	1	Blais JJ	2008	J Youth Adolescence	1
4		0.01	1	Beran	2005	J Educ Comput Res	1
2		0.01	1	Appelman DL	1995	Law Internet	3

*Burst years correspond to years of significant citation activity

Six publications, including Raskauskas and Stoltz [32]; Kowalski and Limber’s as well as Smith et al.’s work from 2007 to 2008 [5] were one of the earliest studies that noted the prevalence and nature of *electronic bullying*, *victimization*, *and perpetration* among American pupils [5,32,33]. Dehue et al. [34] focused on youngsters’ experience of cyberbullying as well as their parents’ perception about it. They found that parents do set rules for the use of the Internet for their children but are not conscious of their perpetrating behavior and also underestimate victimization experiences [34]. Slonje and Smith reported four types of cyberbullying—by text message, email, phone call, and video clip—and emphasized that bullying by video clips is perceived as most negative in the society, and most of the pupils tell their school friends about their experiences and not their parents [35]. Erdur-Baker explained the risky use of the Internet and its association with cyberbullying in Turkey and was one of the rarer studies conducted outside the US [36].

Tokunaga provided synthesized critical review evidence of cyberbullying and provided an integrative definition of cyberbullying, differentiated it from traditional bullying, and linked it with serious psychosocial and affective problems [20]. His work also outlined the areas of concern in research on cyberbullying and provided a framework for future research [20]. In a similar vein, Junon and Gross [37] reported patterns of cyberbullying and their association with social anxiety among school going children [37]. Hinduja and Patchin provided the earliest link of cyber-aggression and increased risk of suicide [4]. Ybarra et al. [38] associated cyberbullying to rule-breaking behavior and aggression in real life in a dose-dependent manner [38].

Two studies focused on the development of the most widely used psychometric questionnaires in cyberbullying. Calvete et al.’s [39] work was the earliest work that led to the development and validation of the Cyberbullying questionnaire for profiling aggressors and cyberbullies [39]. They also reported that the use of proactive aggression, justification of violence, exposure to violence, and less perceived social support of friends was prevalent among cyberbullies [39]. A cyber-dating abuse questionnaire assessed two latent constructs: direct aggression among romantic partners and monitoring control, such as the use of personal passwords [40]. Another of the two studies reported teen dating abuse using an online medium and online sexual solicitations in chat rooms and its risk factors including using chat rooms, using the Internet with a cell phone, talking with people met online, sending personal information to people met online, talking about sex online, and experiencing offline physical or sexual abuse [41,42].

### Domains of research: Cluster analysis

**Fig 6:**
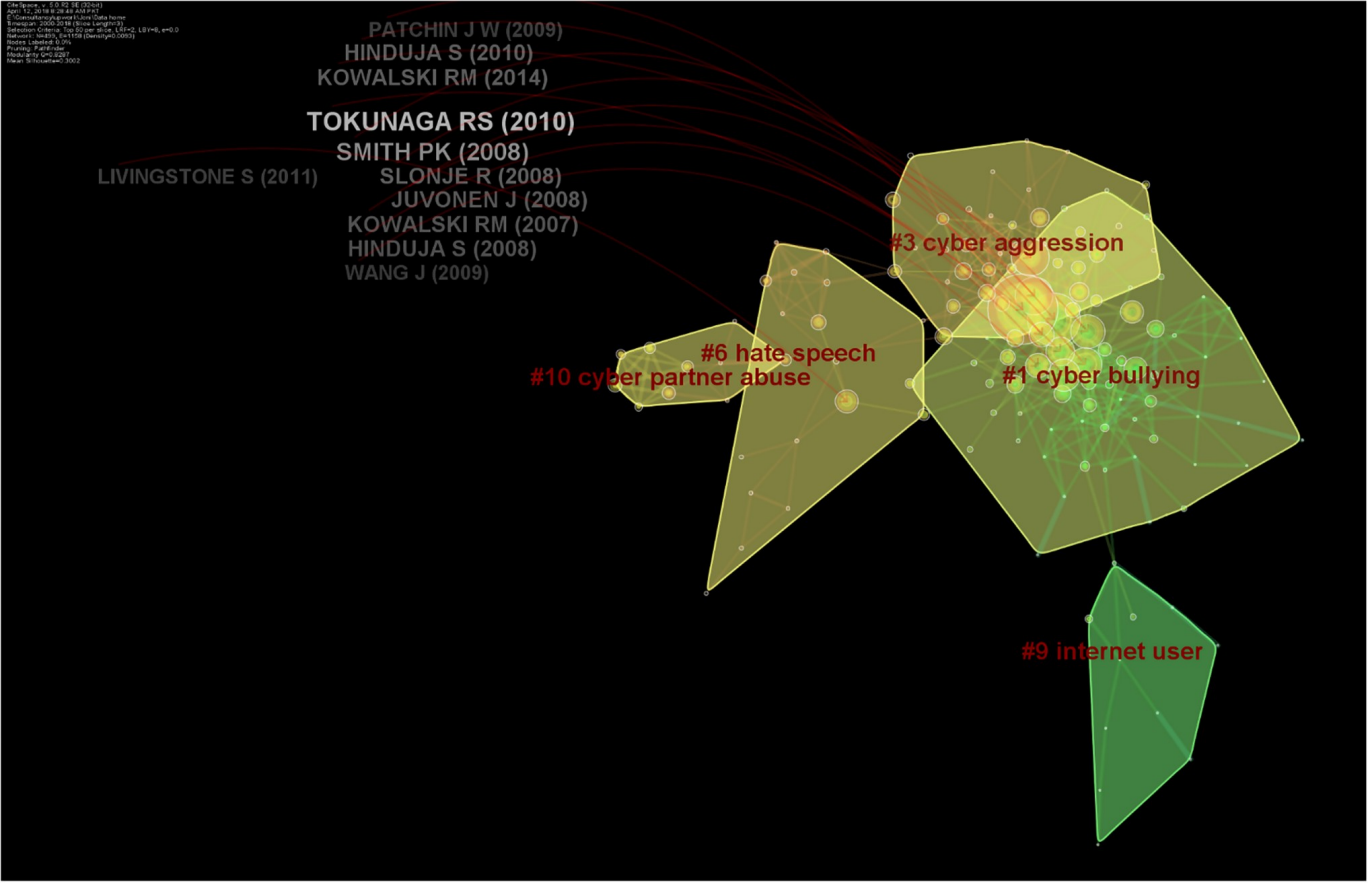
Clusters of research from the year 2000 to 2018.

**Fig 7:**
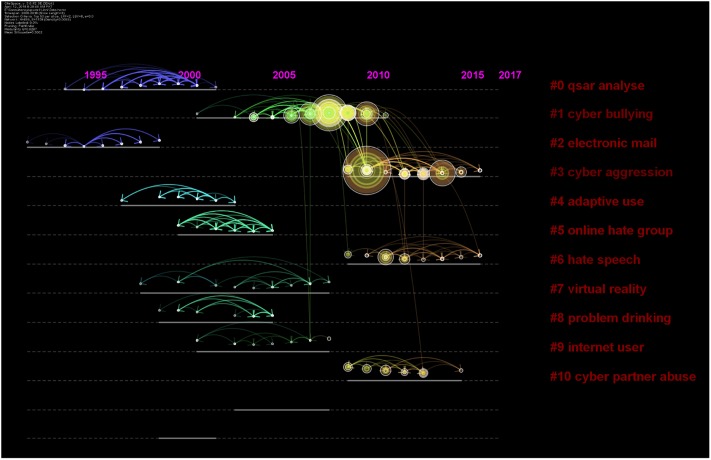
Timeline view depicting clusters of research arranged on a horizontal timeline from 2000 to 2018.

A total of 101 clusters of research emerged in the cluster analysis (Fig 6). These clusters were given names according to four methods: Latent Semantic Indexing (LSI), Term Frequency * Inverted Document Frequency (TF*IDF), loglikelihood ratio (LLR), and Mutual Information (MI). We report in parentheses which method was used to derive the name for a given cluster; generally, it is not important to report all of them, as the outputs of each method were not always sensical. Detailed information regarding the top 10 clusters and their timelines have been presented as Figs 6 and 7. This analysis was based on 499 nodes and 906 lines or edges and yielded modularity of 0.86.

### Clusters on cyberbullying

Five clusters focused on the theme of cyber-bullying. The first meaningful cluster (n = 48, silhouette value = 0.91) emerged as a social networking site as per TF*IDF, cyberbullying, internet harassment and sexual harassment and cyberbullying experience (MI) in 2006 (mean year of publication of included studies). In other words, there were 48 research articles with a similar theme that could be presented with the cluster title of “social networking site” by the TF * IDF method. These 48 articles were placed in this cluster because all of them were cited by a similar group of publications, thus, representing a co-citation relationship. The most cited of this group was Mishna [43] who investigated cyberbullying behaviors among Canadian adolescents. They reported that bullying perpetrators perceived themselves as funny, popular, and powerful, albeit feeling guilty as well [43]. The second meaningful cluster included 48 studies with a silhouette value of 0.88 in 2011. It was named as general strain theory (TF*IDF), cyberaggression (LLR), and Australian youth (MI). The most active citer was Kowlaski et al. [44], who reported cyberbullying behavior among college students across multiple domains of life [44].

Cyberbullying and utilization of routine activity theory were discussed in the seventh cluster with 15 members, a silhouette value of 0.99 and the mean year 2004. It was termed as social networking site by TF*IDF method, internet user, utilizing routine activity theory, potential factor by LLR method, and case study by MI method. The most active citer of this cluster was Marcum et al. [45], who provided causal reasoning for cyber-victimization utilizing the framework of routine activity theory [45]. This theory posits that victimization requires three factors: the presence of a likely offender, a suitable target, and the absence of a capable guardian [45].

The 12^th^ cluster focused on the association of spending time in online communities (TF*IDF) with the mental health of adolescents and caregiver-child relationships (LLR and MI). This cluster included seven papers with a silhouette value of 1.00 in 2000. The most active citer of this group was Ybarra et al. in 2004, who focused on Internet harassment and its association with quality of child-caregiver relationship [46]. The 16^th^ cluster reported papers on an educational and artistic intervention to prevent cyberbullying. It was termed as virtual drama, the emergent narrative approach, and anti-bullying education (TF*IDF, MI, LLR), and emerged in 2005 [47]. The most active citer, Aylett et al. [47] presented evidence for virtual educational software to prevent cyber-bullying.

### Clusters of sexual solicitation and intimate partner violence

A total of three important clusters focused on the theme of sexual solicitation, dating abuse, and intimate partner violence. The third cluster focused on social support (TF*IDF) sexual solicitation via electronic mail; seeking human service; social support (LLR and MI) and included 44 papers. The most active citer was Finn (2000), who described the dangers involved when women seek human services on the internet [48]. This cluster emerged in the year 1998, highlighting early years of research.

The sexual solicitation was the focus of another cluster with 17 papers and a silhouette value of 0.94, emerging in the year 2012. It was termed as extent, situational factor (LSI); hate speech, network site, and online sexual solicitation (LLR, MI). It focused on the abuse of minors as well as online exposure among the youth as evident by its most active citers [49].

The tenth cluster focused on intimate partner violence by utilizing routines activity theory, comprising ten papers in the year 2011 and a mean silhouette value of 0.99. It was labeled as information security; the extent of cyberbullying behavior (TF*IDF), cyber partner abuse, systematic review, routine activities theory, and empirical study (LLR, MI). The most active citer for this cluster was Arntfield (2015), who proposed a new framework for understanding cyber victimology using the Routines Activity Theory Framework [50]. The author stressed the role of victims as both a facilitator and factor for predation [50]. The terms “systematic review” and “empirical study” refer to the study designs utilized by studies in these clusters.

### Clusters on deep learning & automation

Deep learning and automation were studied in two important clusters. The fourth cluster focused on cyber defense (TF*IDF) and adaptive use and network-centric mechanism (LLR) and emerged in 2000. The most active citer was Atighetchi in 2000, whose work focused on defending against network-based attacks, and development of technologies augmenting an application’s resilience against hackers [51]. The 20^th^ cluster revealed deep learning models and text classification as a viable source for identification of hate speech on Facebook groups in 2016 with a silhouette value of 1.0. The papers by Agrawal et al. [52] and Pitsilis et al. [53] were the most common citers of these clusters. Pitsilis et al. [53] proposed recurrent neural network models to discern hateful content on social media utilizing user-related information such as their tendency toward racism and sexism [53], while Agrawal et al. [52] showed that previous algorithms aiding in detection of cyberbullying have bottlenecks: specific platform, a specific topic of bullying, and thirdly, reliance on handcrafted features of the data. They proposed that deep learning models are viable in all of these situations [52].

### Clusters on extremist & online hate groups

This cluster (#5) emerged in the year 2002 and included 18 research items. It was named as extremist groups and mining communities (TF*IDF); online hate group, mining communities, attack tolerance (LLR, MI). The most active citing paper of this cluster was published by Chau et al. [10], who emphasized the importance of analyzing the trends of online hate communities and terrorist groups who share their ideologies to recruit new members. They proposed network analysis and mining techniques as important weapons in this arena [12]. The 14^th^ cluster revealed the use of discourse theory and critical theory as a framework for studying online Islamophobia (TF*IDF, MI). This cluster also had studies focusing on feminism and compensatory manhood (LLR). The most active citer reported harassment and misogyny in online sexual market places and dating websites such as Tinder [54]. The cluster also includes papers on automatic identification and classification of misogynistic languages on social media using NLP and machine learning methods [55]. Moreover, a paper on Islamophobia revealed 11 fake Facebook pages run by Danish citizens posing as Muslims threatening to kill and rape Danish citizens, termed as platformed antagonism [56].

### Keyword analysis

**Fig 8:**
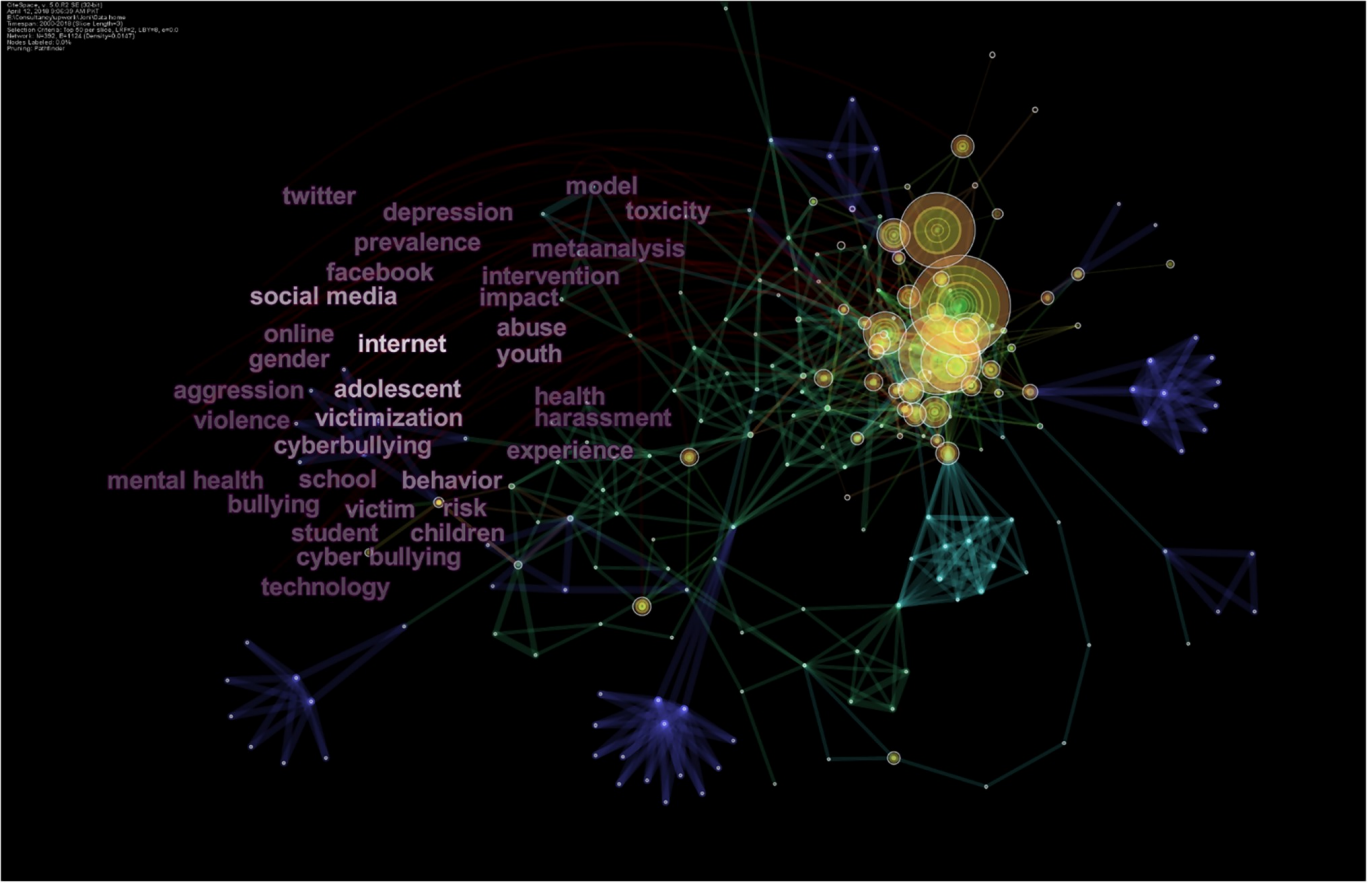
Co-occurrence of keywords.

Furthermore, we used keywords from titles, abstracts, and keywords sections of the research papers to construct keyword co-occurrence networks (see Fig 6). Co-occurrence and frequency of occurrence of keywords provide a snapshot and a reasonable description of trends of research in a specific area [26]. Also, analysis of burst items provides short periods of significant activity in a particular domain or an emerging topic and research frontier [26]. Fig 6 presents the most frequently cited keywords, with larger rings presenting significant keywords. According to it, Internet, adolescents, victimization, social media, Facebook, Twitter, experience, gender, children, victim, victimization, youth, school, toxicity, abuse, and risk most frequently occurring items cited at least 90 times in the literature. Table 4 lists the top 25 cited keywords, and Fig 8 presents co-citation relationship between keywords.

**Table 4:** Top cited keywords.

Citations	Keyword	Mean year of citation of keyword
385	Internet	2002
305	Adolescent	2004
273	Social media	2012
241	Victimization	2007
178	Cyberbullying	2012
176	Behavior	2004
167	Youth	2004
143	Abuse	2003
118	Risk	2004
116	Children	2004
115	Facebook	2012
115	Victim	2002
114	Toxicity	2000
110	Gender	2007
104	Experience	2003
104	Online	2009
103	Impact	2004
97	School	2004
97	Twitter	2012
95	Cyber bullying	2007
94	Prevalence	2007
92	Depression	2004
91	Student	2007
87	Aggression	2004
82	Intervention	2007

**Fig 9:**
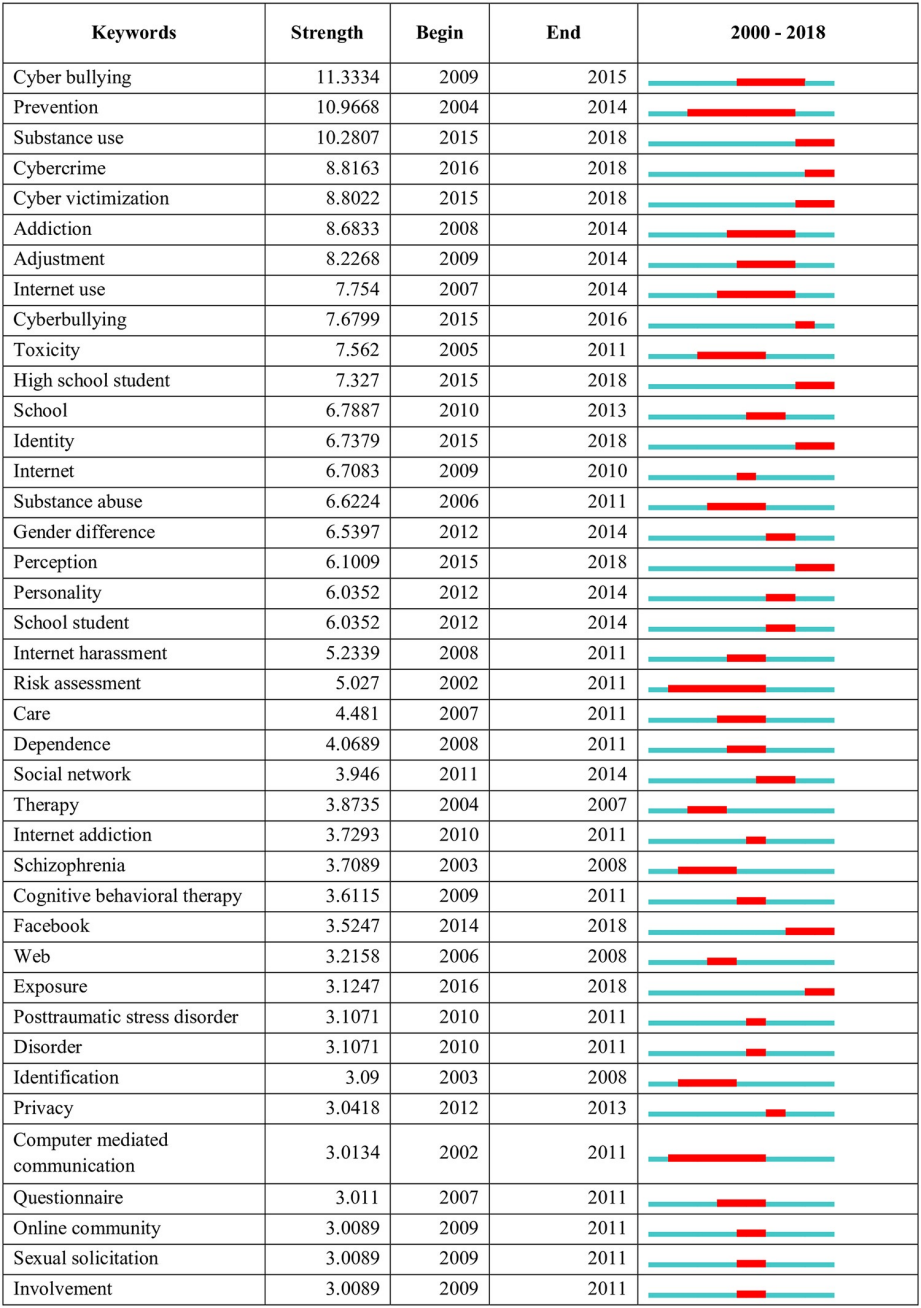
Top keyword bursts.

When burst items analysis was conducted, a total of 53 burst items were identified (see Fig 9). The time interval of the scientometric analysis (2000–2018) has been depicted as a blue line and the period that represents the burst activity, as a red line [26,57]. It presented four main themes of research hotspots in this field, including:

1. Cyberbullying: this hotspot focuses on the pattern of cyberbullying such as cyber-victimization; cyber-bullying, harassment; privacy intrusion; sexual solicitation and involvement.
2. Social media platforms: focused on online communities and specific social media platforms for detection and prevention of hate speech using deep learning and automation.
3. Co-morbid disorders: this hotspot is characterized by keywords such as addiction; substance use; post-traumatic stress disorder; and Internet addiction, citing the importance of co-morbid mental health symptoms among aggressors and victims of cyberhate.
4. Profiling of aggressors and victims: It was characterized by keywords such as identity; school student; personality; gender differences; and identification and risk assessment. These citation bursts exhibit increased research focused on psychological characteristics of both the aggressor and victims. This group also stratifies the population based on their demographic characteristics and increased risk of bullying behaviors.

## Discussion

### Summary of results

The present study highlights the trends of research in the field of OHR. It revealed several clusters of OHR, innovative techniques to detect hate speech, sexual solicitation, exposure to pornography, Islamophobia, misogyny, and cyber-bullying along with its effects among the youth. The US was the lead contributor to this field of research, and our analysis also revealed a clear dominance of Western universities as well as funders from North America, Europe, and China. This global dominance and a higher share of Western institutions have been noted in several empirical investigations [58–60]. Moreover, our analysis revealed a major contribution from psychology-related fields, spanning across the study of human behavior, psychological profiling of aggressors and victims, and co-morbid disorders such as depression and Internet addiction or pathological Internet use, as well as the association between offline and online bullying behaviors. These studies are highlighting the negative consequences of online hate, such as the increased risk of suicide among the victims of cyberbullying [35–37,43]. Overall, there has been a significant increase in publication and citation trend in OHR after the year 2005, which coincides with the proliferation of social media platforms and the Internet becoming a central arena for public and private discourse.

### Strengths, limitations, and future work

There are several strengths and limitations to this study. This is a first concerted effort to map the research activity on online hate. In contrast to previous studies designed as qualitative content analyses or literature reviews on a restricted topic, this study provides a broader analysis of publications of online hate. However, there are a few limitations to this study. Co-citation analyses is a quantitative technique to map research output in a field, and there are several other indicators such as the number of citations accrued or quality of a research article [61]. The role of citation frequency alone to map most influential studies has been long debated [61].

Moreover, while our analysis revealed a major contribution from psychology-related fields, this high representation of psychology-related contributions may be due to several reasons; for instance, the choice of WOS core as the database. Its coverage may be geared towards health and social science disciplines rather than engineering or computer sciences [62], thereby excluding some relevant research from these fields from the analysis. It may also be because there has been a mushroom growth and development in psychology-related publications, interdisciplinary and collaborative networks, as well as higher citation rates, took place in this domain. While we defend the choice of the WOS core database because it is one of the few databases yielding records for cited references [25,28] and embodying a curated collection of over 20,000 peer-reviewed publications pertaining to 250 disciplines in science, social sciences, and humanities [25,28], thereby being accessible for scientometric analyses, we acknowledge that there is a body of OHR literature that is not included in our analysis due to sampling limitations. Future research should aim at replicating or extending this study by accessing literature from other databases, such as ACM Digital Library.

### Implications for research and practice

The main lessons learned from this scientometric analysis are as follows:

1. Most of the publications originate from the discipline of psychology and psychiatry with recurring themes of the prevalence of cyber bullying, psychiatric morbidity, and psychological profiles of bullies and victims, particularly among the youth. In later years, there was some focus on dating violence and harassment of women. The main implication is that policy makers, and funders need to shift their focus on other fields, such as intervention and implementation sciences to design both technological and non-technological solutions to identify and curb online hate.
2. Almost all the influential studies have been conducted in the context of high-income countries. Research is needed in low and middle-income countries to justify the generalizability of OHR findings as well as to produce culturally applicable interpretations.
3. As far as we are aware of, this is the first concerted effort to map global research output regarding OHR, spanning across scientific disciplines such as psychology, computer sciences, and the social sciences. However, the dominance of psychology related publications may have skewed the overall results. For this reason, we also encourage discipline-specific scientometric studies because most of the studies published to date were i) discipline or population-specific, ii) simplistic literature reviews, and iii) lacked systematic search process and iv) reproducible data science techniques.

In conclusion, the increase in OHR is a reaction to the increased occurrences of hate speech, in all of its various forms, on the many social media and other online platforms. Online hate speech is, obviously, a complex societal problem that intersects many aspects of everyday life. The cross-disciplinary and multifaceted nature of OHR as a field of research is a witness to the complex issue of online hate. The findings from research so far hint at the need for both technology and non- technology approaches to address this increasingly pressing societal issue.

## Supporting information

S1 Dataset All data associated with this study have been provided as a supplementary file named data supplement.zip. (ZIP)
